# Development of Hybrid Deep Learning Systems Combining Convolutional Neural Networks and Vision Transformers for Medical Ultrasound Image Classification

**DOI:** 10.3390/diagnostics16213640

**Published:** 2026-11-04

**Authors:** Hasan Ucuzal, Cemil Colak, Emek Guldogan

**Affiliations:** Department of Biostatistics and Medical Informatics, Faculty of Medicine, Inonu University, 44280 Malatya, Turkey; cemilcolak@yahoo.com

**Keywords:** deep learning, convolutional neural network, vision transformer, hybrid architecture, thyroid nodule classification, explainable artificial intelligence, medical image analysis

## Abstract

**Background**: Accurate classification of thyroid nodule ultrasound images is clinically significant. This study evaluated whether hybrid architectures combining the local feature extraction of convolutional neural networks (CNNs) with the global context modelling of vision transformers (ViTs) outperform each category in isolation. **Methods**: Two public datasets (6905 images; 3331 benign, 3574 malignant) were used. Eleven ImageNet-pretrained architectures were compared: CNN-based (ResNet-50, EfficientNet-B4, ConvNeXt), ViT-based (Swin Transformer, DeiT, PVTv2, XCiT) and hybrid (ConViT, CoaT, MobileViT, HuviT). Backbones were frozen and only the classification head was trained; HuviT’s new projection and encoder remained frozen at random initialisation. Wilson and Newcombe 95% confidence intervals (CIs) were computed; explainability used gradient-weighted class activation mapping (Grad-CAM). **Results**: ConViT achieved the highest accuracy (83.30%; 95% CI 80.91–85.45) and an area under the receiver operating characteristic curve (ROC-AUC) of 89.94% (95% CI 87.8–91.7). ConvNeXt ranked second (82.63%) but had the highest ROC-AUC (90.02%; 95% CI 87.9–91.8); the intervals overlapped. HuviT reached 76.35%. Grad-CAM for a representative case highlighted lesion areas. **Conclusions**: While ConViT achieved the highest performance on threshold-based metrics, these findings suggest the potential of specific hybrid designs rather than establishing general superiority of hybrid architectures. Given the retrospective design and the partial association between image source and class label, prospective external validation is warranted.

## 1. Introduction

The volume of high-dimensional medical imaging data generated by magnetic resonance imaging (MRI), computed tomography (CT), digital pathology, and ultrasound (US) has grown sharply over the past decade [1]. Managing this growth while maintaining diagnostic precision has made automated analysis systems a practical necessity rather than a convenience [2]. Deep learning, in particular, has reset expectations: models trained on large image repositories can match or approach clinician-level accuracy on well-defined tasks [3], outperforming classical machine-learning pipelines built on handcrafted features [4], and their deployment as decision-support tools is an active area of research [1,2].

CNNs have been the dominant architecture for visual recognition in medicine. Their inductive biases toward local spatial structure, achieved through weight sharing and pooling, translate efficiently into feature hierarchies that capture edges, textures, and shapes [5]. A fundamental limitation, however, is that long-range dependencies can only be captured by stacking many layers, since a neuron in any given layer receives input only from a spatially local neighbourhood [6]. The self-attention mechanism introduced by transformer architectures addresses exactly this constraint [7]. When adapted to images by treating fixed-size patches as sequence tokens, transformers can model relationships across the full spatial extent of an image in a single operation [8]. This global receptive field makes ViTs particularly appealing for tasks where understanding image-wide structure matters.

The practical trade-off is well established: ViTs tend to require larger training datasets to generalise from scratch and typically incur higher computational costs than CNNs of comparable parameter count [9,10]. In medical imaging, where labelled data are expensive to obtain, this can be a genuine bottleneck. Hybrid architectures, which selectively combine convolutional layers for local feature extraction with transformer layers for global context modelling, offer a middle path [11]. Evidence from general computer vision suggests these hybrids can outperform either class alone, yet the optimal integration strategy, training regime, and generalisability across imaging modalities remain active research questions.

Thyroid nodule assessment is a clinically demanding test case. Ultrasonography is the primary screening tool, but reliable differentiation of benign from malignant lesions requires evaluating echogenicity, margin irregularity, microcalcification, and vascularity, features that span both fine local texture and global lesion context. Interobserver variability among radiologists is well documented, and second-opinion workflows are standard in many centres. A model that identifies malignant nodules with high recall while keeping false-positive rates low might help reduce unnecessary biopsies and support earlier treatment decisions.

Against this background, the present study pursued two goals: (1) a systematic head-to-head comparison of eleven architectures spanning three paradigms on a thyroid ultrasound dataset; and (2) the introduction and evaluation of HuviT, a novel hybrid architecture combining a pretrained ResNet-50 backbone with a multi-layer transformer encoder. The evaluation extended beyond aggregate accuracy to include class-level metrics, interval estimates for the principal performance measures, and visualisation by explainable artificial intelligence (XAI), aiming for results that are both technically rigorous and clinically interpretable.

## 2. Background

### 2.1. CNN Architecture

**Figure 1:**
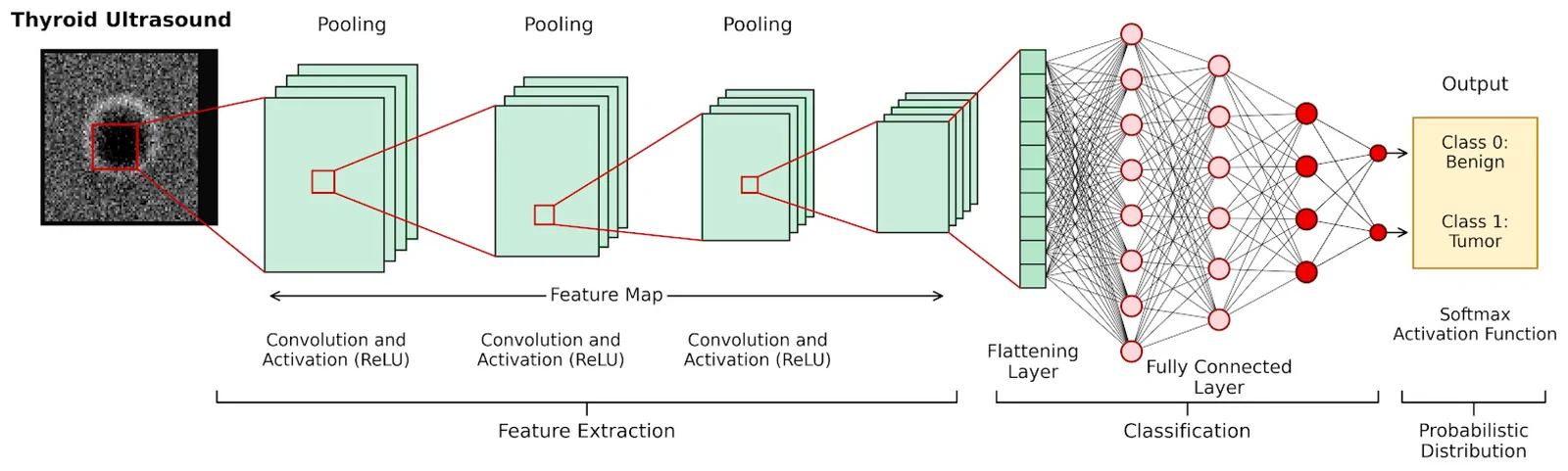
Schematic diagram of the CNN architecture illustrating convolutional, pooling, activation, and fully connected layers. Abbreviations: FC, fully connected.

CNNs learn visual features by applying learnable filters that slide across an input image and produce feature maps summarising local responses to patterns such as edges and textures. As the network deepens, these local representations are combined hierarchically so that higher layers encode progressively abstract concepts. Key components include convolutional layers for feature extraction, pooling layers for spatial downsampling and translational invariance, rectified linear unit (ReLU) activation, and fully connected layers that map learned features to output predictions [5,12,13,14]. The inductive bias toward local connectivity and weight sharing makes CNNs sample-efficient and computationally tractable for medium-scale datasets; however, inferring global relationships requires the indirect cumulation of many local operations, which limits the capture of long-range spatial dependencies [6]. A schematic overview of a standard CNN architecture is provided in Figure 1.

### 2.2. ViT Architecture

The Vision Transformer adapts the transformer encoder to image data by dividing an image into a grid of non-overlapping patches (typically 16 × 16 or 32 × 32 pixels) and linearly projecting each flattened patch into an embedding space [8]. A learnable class token is prepended, and positional embeddings are added to each token to encode spatial order. The resulting sequence passes through transformer encoder blocks comprising multi-head self-attention (MHSA) and feed-forward multilayer perceptron (MLP) sub-layers with layer normalisation and residual connections [7]. The MHSA mechanism computes pairwise attention weights across all tokens simultaneously, so any patch can attend to any other patch regardless of spatial separation. This global receptive field from the first layer onward is the chief advantage of ViTs for structurally complex images. The primary costs are quadratic scaling of attention with sequence length and a pronounced reliance on large pretraining corpora for adequate generalisation [9,10].

### 2.3. Hybrid Architectures

Hybrid CNN–Transformer architectures aim to capture local spatial features through convolutional operations while relying on self-attention to model global dependencies. They can be classified by integration strategy: sequential hybrids pass CNN-derived feature maps directly into a transformer encoder; parallel hybrids process the same input with both components simultaneously and fuse the outputs; and transformational hybrids treat each stage as semantically re-encoding the data so that later components receive qualitatively different representations rather than filtered versions of the input. Within this space, notable architectures include DeiT, which trains a ViT student using knowledge distillation from a CNN teacher to reduce the data requirement [15,16]; ConViT, which initialises self-attention with convolutional inductive biases that gradually open toward global attention during training [17]; CoaT, which integrates depth-wise convolutions into attention layers and links feature maps across spatial scales [18]; Swin Transformer, which computes attention within shifted local windows to produce a hierarchical feature pyramid [19]; and MobileViT, a lightweight hybrid designed for resource-constrained deployment [20]. Additional hybrid designs include DETR (DEtection TRansformer), which applies a transformer decoder to learnable object queries for end-to-end detection [21], and Mobile-Former, which bridges MobileNet and transformer through a bidirectional cross-attention bridge [22]. Table 1 summarises the taxonomy of hybrid categories, their structural characteristics, advantages, limitations, and representative application domains.

The rationale for hybrid designs is particularly strong in medical imaging. Lesion boundaries, textures, and microstructural details depend on local convolutional processing; the relationship between a lesion and surrounding tissue, or the global distribution of calcification across an image, requires long-range attention. Hybrid models address both levels simultaneously, and they are often reported to generalise better than pure ViTs on the smaller datasets typical of clinical research. This pattern has been observed across medical imaging tasks beyond thyroid ultrasonography: a hybrid pipeline that couples a pretrained CNN feature extractor with a support vector machine classifier outperformed a fine-tuned CNN in brain tumour classification [23], and a small-scale experiment on CIFAR-10 reports that adding convolutional components to a ViT improves its accuracy when training data are limited [24], because the convolutional component provides regularisation that reduces dependence on massive pretraining [11,17].

In recent years, work has begun to address the local–global trade-off specifically in thyroid ultrasonography. A dedicated review of vision-transformer methods for ultrasound image analysis reports accumulating evidence that transformer and hybrid designs improve accuracy and efficiency across ultrasound classification, detection and segmentation tasks, while identifying a set of open challenges that still stand in the way of clinical translation [25]. Jerbi et al. combined convolutional feature extraction with vision transformers and generative adversarial augmentation for benign–malignant nodule classification [26], and İnan et al. proposed a hybrid ResNet-based U-Net pipeline that couples automatic nodule segmentation with multi-class classification in a cohort of 880 patients [27]. More recent designs integrate the two paradigms at the feature level rather than sequentially: LGF-Net runs a wavelet-transform and deformable-convolution CNN branch in parallel with an aggregated-attention transformer branch and fuses the two representations [28], while other groups have paired vision transformers with Wasserstein generative adversarial networks with gradient penalty in order to mitigate class imbalance in thyroid ultrasound cohorts [29]. Three features of this literature motivate the design of the present study. Comparisons are typically restricted to two or three architectures evaluated against a single baseline, so the relative standing of the CNN, ViT and hybrid paradigms under one protocol remains unestablished. The reference standard and the data source differ between reports, which makes headline accuracies difficult to compare. Finally, reported accuracies are rarely accompanied by interval estimates, so it is often unclear whether a stated ranking would survive resampling.

## 3. Materials and Methods

### 3.1. Dataset

Two publicly available thyroid nodule ultrasound datasets were combined, yielding 6905 images in JPEG and PNG format: 3331 labelled as benign and 3574 as malignant. The base dataset was TN5000 [30], which contributes 5000 images (1426 benign, 3574 malignant) and is therefore markedly imbalanced towards the malignant class. To correct this imbalance, a further 1905 benign images were added from a publicly released Kaggle collection [31], bringing the pooled dataset close to class balance (48.2% benign, 51.8% malignant). No malignant images were taken from the second source. The data descriptor for TN5000 reports 3572 malignant and 1428 benign cases, whereas a direct count of the class labels in the published PASCAL VOC annotation files (the <name> field of each <object> element, in which 1 denotes a malignant and 0 a benign nodule) yields 3574 malignant and 1426 benign. The discrepancy therefore concerns two images in each class, does not alter the class balance of the dataset materially, and most plausibly reflects a small difference between the cohort described in the text and the files ultimately deposited. The counts obtained from the annotation files are adopted throughout this study, because they correspond exactly to the files that were read, decoded and partitioned by the pipeline. The two sources also differ in provenance, and this bears directly on the interpretation of the results. TN5000 was assembled at a single institution, the Cancer Hospital of the Chinese Academy of Medical Sciences, from B-mode examinations acquired on GE Logiq E9 and S7 systems at 5–12 MHz or 8–15 MHz; its heterogeneity is therefore one of probe, operator and acquisition setting rather than of centre [30]. The provenance of the Kaggle collection is not documented by the uploader: the release carries a one-line description and is accompanied by no data descriptor, acquisition protocol or list of contributing centres [31]. The pooled dataset should accordingly be described as two-source rather than as multi-centre, and the generalisability claims made in this paper are framed on that basis. Because the two sources were pooled before partitioning, the training, validation and test subsets each contain images from both.

To evaluate possible overlap between the two sources, a cross-dataset image deduplication was performed between TN5000 (*n* = 5000) and the Kaggle collection (*n* = 1905). Both cryptographic hashing (SHA-256, for exact byte-level identity) and perceptual hashing (pHash distance threshold ≤5, for structural and visual similarity under noise and compression) were computed across all cross-dataset image pairs. No exact or near-duplicate pair was found, so overlap of identical or near-identical images between the two sources can be excluded.

#### 3.1.1. Reference Standard and Labelling

The two sources do not share a reference standard. In TN5000, the benign/malignant label of every image is anchored in fine-needle aspiration (FNA) biopsy and the associated pathological result: nodule location was annotated first by junior radiologists and then verified image by image by a senior radiologist with more than thirty years of experience, and the dataset authors state explicitly that the class label is determined by FNA and pathology rather than by a Thyroid Imaging Reporting and Data System (TI-RADS) category, which they identify as the principal weakness of earlier public thyroid datasets [30]. The reference standard for the 1905 benign images drawn from the Kaggle collection cannot be established from the information the uploader provides; the release states neither whether the labels derive from cytology, histopathology, imaging follow-up or expert ultrasound assessment, nor by whom they were assigned [31]. The benign class of the pooled dataset is therefore labelled to a mixed standard that is undocumented for 57.2% of its images, whereas every malignant image carries a pathology-anchored label.

#### 3.1.2. Source–Label Association and Its Magnitude

Because all 3574 malignant images originate from TN5000 while 1905 of the 3331 benign images (57.2%) originate from the second source, image source is partly associated with the class label. The magnitude of the resulting confounding can be bounded exactly. Consider a hypothetical classifier that uses no nodule morphology whatsoever but recognises only which dataset an image came from and applies the majority label of that source: it would assign ‘benign’ to every Kaggle image (1905 of 1905 correct) and ‘malignant’ to every TN5000 image (3574 of 5000 correct), achieving (1905 + 3574)/6905 = 79.35% accuracy on the pooled data. This figure is an upper bound on what source identity alone can contribute, and it exceeds the accuracy obtained here by five of the eleven architectures. It does not show that the models exploited source cues, but it does show that overall accuracy on the pooled test set cannot by itself distinguish the two explanations. A source-stratified sensitivity analysis would therefore be informative.

#### 3.1.3. Case Mix, Nodule Composition and Multinodular Glands

No selection was applied based on nodule composition, echogenicity, calcification or number: every image released in the two sources was used. No sonographic subtype was therefore excluded by design, and the evaluated cohort is expected to contain solid, cystic and mixed nodules, lesions of differing echogenicity, and nodules with macro- or microcalcification in whatever proportions the sources happen to hold. This cannot be verified, however, because neither source distributes per-nodule descriptors of composition, echogenicity, calcification or vascularity, so the statement is an inference from the absence of exclusion criteria rather than an observation. The only inclusion criteria are those inherited from the TN5000 curation, namely that each image must contain at least one discernible thyroid nodule and that only pure B-mode images without contrast enhancement were retained [30]. Neither source records whether an examination was performed in a multinodular gland. With these public data, it is consequently not possible to report performance separately for calcified, anechoic or predominantly cystic nodules, nor to establish whether accuracy is maintained in multinodular goitre, where the operator must select a target lesion and adjacent nodules may lie within the field of view. The models classify single images and are not designed to arbitrate between several nodules in the same gland. TN5000 was assembled at a tertiary oncology centre, so the malignant fraction of the pooled data (51.8%) lies far above the malignancy rate of roughly 5–15% reported for thyroid nodules encountered in routine practice and used as the basis of current management guidance [32].

### 3.2. Data Preprocessing

All images were resized to 224 × 224 pixels to match the input dimensions of ImageNet-pretrained models. Normalisation used ImageNet channel statistics (means [0.485, 0.456, 0.406]; standard deviations [0.229, 0.224, 0.225]). Because the original images were already in grayscale format, no colour-space conversion was performed; channels were replicated as necessary to produce three-channel inputs.

### 3.3. Data Augmentation

Six augmentation operations were applied on-the-fly during training to limit overfitting and increase effective data diversity: (1) horizontal flipping; (2) random rotation within ±10°; (3) brightness jitter of ±30%; (4) contrast jitter of ±30%; (5) random crop retaining 80–100% of the image area followed by resize; and (6) additive Gaussian noise. These transformations simulate realistic probe position variation and scanner-to-scanner differences without generating anatomically implausible views. Augmentation was applied only to the training partition.

### 3.4. Data Partitioning

The full dataset was randomly split into training (70%), validation (15%), and test (15%) subsets using a fixed random seed of 42, yielding 4833 training images, 1036 validation images, and 1036 test images. Class proportions were preserved across all partitions (test set: 500 benign and 536 malignant images). The test set was held out entirely throughout training and hyperparameter selection, accessed only for final performance reporting.

The split was performed at the image level. Neither source distributes patient or nodule identifiers: TN5000 was anonymised before release, and the dataset authors state that names, sex, age, pathology numbers and all other identifying information were erased in order to protect patient privacy [30], while the Kaggle collection supplies no metadata whatsoever [31]. Patient-level or nodule-level partitioning was therefore not achievable, and the same constraint applies to the official train–validation–test split distributed with TN5000, which is itself a random image-level split in a 7:1:2 ratio [30]. Two features of the TN5000 curation limit, without eliminating, the resulting leakage risk. The 5000 released images were selected from a pool of more than 30,000 under the rule that only one representative image per imaging perspective per patient is retained, a step the dataset authors describe as intended precisely to prevent data leakage and to permit partitioning at the patient level [30]. Residual risk nevertheless remains where a single patient contributed images from more than one perspective, since such images would be correlated and could be distributed across the training and test partitions; the same applies, with unknown magnitude, to the second source. Because the identifiers needed to quantify this are not available, the possibility that the reported performance is modestly optimistic relative to a strict patient-level evaluation cannot be excluded, and it is acknowledged among the limitations in Section 5.

Under the random partition described above, the expected source composition of the test set can be stated without access to the file lists. Sampling 1036 of the 6905 pooled images without replacement, the expected number drawn from the 1905 Kaggle images is 1905 × 1036/6905 = 285.8, with a hypergeometric standard deviation of 13.3 and a 95% sampling interval of 260 to 312. Approximately 286 of the 500 benign test images are therefore expected to originate from the Kaggle source and approximately 214 from TN5000. On the test set specifically, the source-only classifier described in Section 3.1.2 would accordingly be expected to reach (286 + 536)/1036 = 79.3% accuracy, which is within 0.1 percentage points of the pooled figure.

### 3.5. Model Architectures

#### 3.5.1. CNN-Based Architectures

ResNet-50 applies residual learning through identity shortcut connections around bottleneck blocks (1 × 1–3 × 3–1 × 1 convolution triplets), enabling 50-layer networks to be trained without gradient vanishing [33]. EfficientNet-B4 scales network depth, width, and input resolution jointly through compound scaling, producing a strong accuracy-to-parameter ratio [34]. ConvNeXt modernises a standard ResNet by adopting design principles from vision transformers (large 7 × 7 depth-wise convolutional kernels, Layer Normalisation, inverted bottleneck structure, and fewer activation functions per block), yielding a purely convolutional architecture that competes with ViTs on large benchmarks [35].

#### 3.5.2. ViT-Based Architectures

Swin Transformer computes self-attention within local non-overlapping windows and introduces cross-window interaction by shifting window positions in alternating layers, producing a hierarchical feature pyramid analogous to that of CNNs [19]. DeiT (Data-efficient Image Transformer) reduces the data requirement of standard ViTs by augmenting the training objective with a distillation token that learns from a CNN teacher’s output, effectively transferring convolutional inductive biases into the ViT student [15]. PVTv2 (Pyramid Vision Transformer v2) combines overlapping patch embeddings, convolutional feed-forward networks, and linear-complexity attention into a strong backbone for dense prediction tasks [36]. XCiT (Cross-covariance Image Transformer) replaces standard token-token attention with cross-covariance attention operating on feature channels rather than spatial tokens, reducing computational complexity from quadratic to near-linear in spatial resolution [37].

#### 3.5.3. Hybrid CNN + Transformer Architectures

ConViT (convolutional vision transformer) incorporates Gated Positional Self-Attention (GPSA) blocks that initialise with strong local inductive biases analogous to convolutions and gradually transition toward global self-attention as training progresses, allowing the model to exploit convolutional regularisation early while learning long-range dependencies later [17]. CoaT (Co-scale Conv-Attentional Transformer) integrates depth-wise convolutions into multi-head attention for implicit positional encoding and adds a co-scale mechanism to exchange information across spatial resolutions [18]. MobileViT combines MobileNet-style convolutional blocks with compact transformer sub-layers optimised for on-device inference [20]. HuviT is the novel two-stage architecture introduced in this study. Its design goal is to obtain global context modelling at a fixed, modest computational cost on top of a widely available convolutional backbone, so that the transformer component can be attached to an existing CNN pipeline without retraining it. A pretrained ResNet-50 backbone, truncated after the final residual stage (average-pooling and fully connected layers removed), produces spatial feature maps of shape [B, 2048, H, W]. These maps are adaptively pooled to a 7 × 7 grid, yielding 49 spatial positions that are flattened into a token sequence and projected to a 512-dimensional embedding by a single linear layer. The sequence is then processed by a four-layer transformer encoder with eight attention heads, a feed-forward dimension of 2048, ReLU activation, post-layer normalisation, residual connections and a dropout rate of 0.1. Global average pooling over the output tokens produces a single 512-dimensional descriptor, which a linear classifier maps to the two output classes. In the implementation used here, only this final linear classifier was trained: the ResNet-50 backbone was frozen at its ImageNet weights, whereas the projection and the transformer encoder, for which no pretrained weights exist, were frozen at their random initialisation. The depth and width of the encoder were fixed a priori rather than tuned: four layers and eight heads keep the transformer component at roughly one third of the total parameter count (see Section 3.6), and the 7 × 7 pooling grid matches the native spatial resolution of ResNet-50 at 224 × 224 input, avoiding interpolation artefacts. Experiments in which the projection and the transformer encoder are trained, together with ablation of the transformer hyperparameters (number of layers, number of attention heads, embedding dimension and feed-forward dimension), are time-consuming and are still in progress; their results will be reported in a separate study. Adaptive pooling also decouples the token count from the input size, so the same encoder can be reused with other convolutional backbones. Because attention operates after the backbone has already compressed the image, HuviT is a sequential rather than an interleaved hybrid; the implications of this choice are examined in the Discussion. The parameter counts of HuviT and their component-wise derivation are given in Section 3.6.

### 3.6. Training Protocol

All eleven architectures were trained under identical settings to ensure that observed performance differences reflect architectural properties rather than procedural variation. Models were initialised with ImageNet-pretrained weights under a transfer learning strategy [38]; the backbone was frozen and only the classification head was updated. This design was deliberate and serves three purposes. First, it isolates the effect of architecture: because every model receives an identical optimisation budget and is trained through the same, deliberately minimal classification head, differences in test performance can be attributed largely to the representational properties of the frozen backbone rather than to unequal fine-tuning dynamics, which vary substantially across CNNs and transformers and would otherwise confound an eleven-way comparison. Second, with fewer than 5000 training images, full fine-tuning of backbones containing tens of millions of parameters carries a high overfitting risk and typically requires architecture-specific learning-rate schedules, layer-wise decay and regularisation, none of which could be applied uniformly. Third, feature extraction with a frozen backbone is the configuration most likely to be reproduced in resource-constrained clinical research settings, so the reported values represent a conservative lower bound on attainable performance. The consequences of this choice for absolute accuracy are addressed in the Discussion. A focal loss (α = 1, γ = 2) was used instead of weighted cross-entropy. Frozen backbones were kept in training mode, so the batch-normalisation running statistics of backbones that contain such layers (ResNet-50, EfficientNet-B4, MobileViT and the ResNet-50 of HuviT) continued to be updated although their weights were not. Shared hyperparameters are listed in Table 2.

To support reproducibility, PyTorch deterministic algorithms and cuDNN determinism were enabled and all random number generators (Python (v3.13.5), NumPy (v2.2.6), PyTorch (v2.7.1+cu118)) were seeded with 42 and re-seeded at the start of every epoch and batch (42 plus the epoch and batch index).

The scope of freezing was the same in kind across the eleven architectures: every parameter of the ImageNet-pretrained backbone was frozen and only the final classification layer was updated, namely a single fully connected layer mapping the backbone’s pooled embedding to two logits, so that the number of trainable parameters equals 2 × (d + 1), where d is the embedding dimension of that backbone (for ConvNeXt, plus the 2d affine parameters of the layer normalisation preceding its linear layer), and differs between architectures only insofar as d does. HuviT follows the same rule. Because its transformer encoder is introduced in this study, no pretrained weights exist for it: the ResNet-50 backbone was frozen at its ImageNet weights, whereas the linear projection to the 512-dimensional embedding space and the four-layer transformer encoder were frozen at their random initialisation, and only the final linear classifier (2 × (512 + 1) = 1026 parameters) was trained (Table 3).

HuviT therefore had a trainable capacity comparable to the other ten models (1026 trainable parameters), but its projection and transformer encoder were never trained and carry random rather than pretrained or learned weights. Its accuracy of 76.35% should be read in this light: it reflects a frozen ResNet-50 representation passed through a fixed random projection and a fixed random transformer, not a trained hybrid design. This is a limitation of the present implementation rather than a property of the architecture, and it is one reason why the HuviT result is presented as a baseline; training the projection and the encoder is the natural next step.

The HuviT row was computed from the model definition: the ResNet-50 backbone contributes 25,557,032 parameters (of which 2,049,000 belong to the fully connected layer that is instantiated but not used in the forward pass), the 2048→512 linear projection 1,049,088, the four-layer post-norm transformer encoder with eight heads and a feed-forward dimension of 2048, 12,609,536 (4 × 3,152,384, each layer including two layer normalisations), and the two-class linear classifier 1026, giving 39,216,682 parameters in total, of which only the classifier (1026) is trainable. The ResNet-50 and EfficientNet-B4 rows are likewise determinate, because those two names fix the configuration: their counts are 23,508,032 + 4098 and 17,548,616 + 3586 respectively, and they are identical whether the weights are taken from timm or from torchvision.

### 3.7. Experimental Setup

All experiments were conducted on a high-performance server with an Intel Xeon Gold 5320 CPU (2.20 GHz, 26 cores), 512 GB DDR4 RAM, an NVIDIA Tesla A40 GPU (10,752 CUDA cores, 48 GB GDDR6, 336 Tensor cores), and 2.5 TB NVMe SSD storage (RAID 0). The software stacks comprised Python (v3.13.5), PyTorch (v2.7.1+cu118), TorchVision (v0.22.1+cu118), PyTorch Hub (DeiT), timm (v1.0.19; XCiT, PVTv2, ConViT, CoaT and MobileViT), Pillow (v11.3.0), NumPy (v2.2.6), OpenCV (v4.13.0), Scikit-learn (v1.7.1), Pandas (v2.3.2), Matplotlib (v3.10.6), and Seaborn (v0.13.2). CUDA and cuDNN provided GPU acceleration. No out-of-memory interruptions occurred during any model’s training run.

### 3.8. Performance Metrics

Model performance was evaluated on the held-out test set using five metrics: accuracy (TP + TN)/(TP + TN + FP + FN); precision TP/(TP + FP); recall (sensitivity) TP/(TP + FN); F1-score 2 × (precision × recall)/(precision + recall); and ROC-AUC, which measures discriminative ability independently of the decision threshold. Precision, recall and F1-score were aggregated over the two classes as support-weighted averages. Here, TP, TN, FP and FN denote true positives, true negatives, false positives and false negatives, respectively. The class-level analysis additionally used specificity TN/(TN + FP), positive predictive value (PPV) TP/(TP + FP), negative predictive value (NPV) TN/(TN + FN) and balanced accuracy, the mean of sensitivity and specificity.

Point estimates alone do not convey the precision of a performance measure, and the differences between the leading architectures in this study are small. Two-sided 95% confidence intervals were therefore computed for every proportion-based metric using the Wilson score interval [39], which is preferred to the normal approximation because it retains nominal coverage for proportions away from 0.5 and at moderate sample sizes. Comparative evaluations of binomial interval estimators support this choice over the Wald interval for the sample sizes used here [40]. ConViT was compared with each of the other ten architectures using an unpaired two-sided two-proportion z-test on the number of correctly classified test images, with Holm’s sequential correction for the ten comparisons. For ROC-AUC, two-sided 95% confidence intervals were computed with Newcombe’s score-type method [41], the counterpart of the Wilson interval for the Mann–Whitney statistic on which the AUC is based; it requires only the AUC and the numbers of malignant and benign test images and keeps the interval within the unit range. Because these intervals are computed separately for each model, they do not account for the pairing of models evaluated on the same test images and are used descriptively rather than for formal pairwise comparison.

### 3.9. Explainable AI Methods

Model decisions were interpreted using Grad-CAM, which generates class-specific heat maps by weighting the final convolutional feature maps with the gradient of the target class score, thereby highlighting the regions most influential for a given prediction [42]. Heat maps were characterised quantitatively by maximum activation, mean activation, and activation area ratio (threshold 0.5), and were qualitatively assessed for anatomical plausibility against accepted radiological criteria for thyroid nodule assessment.

### 3.10. Software Platform

A modular, Streamlit-based web application was developed alongside the research pipeline. The application provides a graphical interface for data loading, preprocessing, augmentation, model training with real-time progress monitoring, evaluation, and XAI visualisation. The software architecture comprises four layers (data management, model, training, and evaluation), each independently configurable and reusable. The system supports export of model weights, performance reports, and heat map visualisations. Screen captures illustrating the workflow from data upload through results reporting are provided as Figures S1–S8 in the Supplementary Materials.

## 4. Results

### 4.1. Overall Performance

Classification performance across all eleven architectures is presented in Table 4. ConViT achieved the highest threshold-based scores: 83.30% accuracy, 83.30% precision, 83.26% recall and 83.30% F1-score, together with a ROC-AUC of 89.94%; the highest ROC-AUC overall was obtained by ConvNeXt (90.02%). Accuracy ranged from 73.75% (MobileViT) to 83.30% (ConViT), a spread of approximately 9.5 percentage points across the full model set.

**Figure 2:**
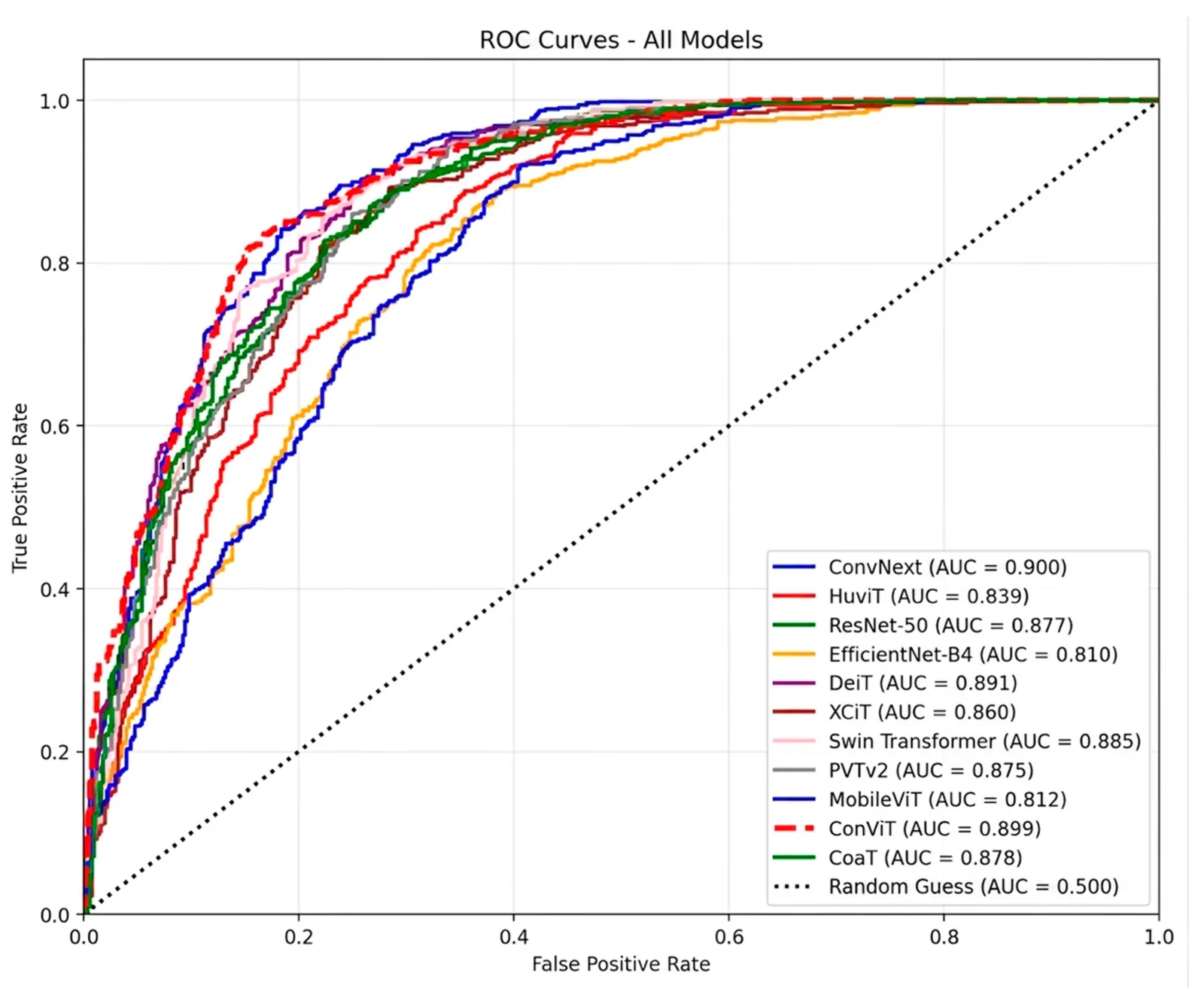
ROC curves for all eleven architectures on the held-out test set. Abbreviations: AUC, area under the curve; ROC, receiver operating characteristic.

Within the CNN category, ConvNeXt ranked second overall with 82.63% accuracy and the highest ROC-AUC across all models (90.02%), a value nominally higher than, and comparable to, that of ConViT (89.94%), despite a marginally lower threshold-based accuracy. The 95% confidence intervals for ROC-AUC were similar for ConViT (0.878–0.917) and ConvNeXt (0.879–0.918) (Table 4), suggesting comparable discriminative ability, while ConViT showed the highest values on the threshold-based metrics. Among pure ViT architectures, DeiT led with 81.18% accuracy and 89.10% ROC-AUC, while PVTv2 and XCiT followed closely. Swin Transformer showed notably higher precision (82.71%) relative to its accuracy (77.80%), a pattern consistent with a decision threshold that yields comparatively few false positives at the cost of more missed cases. Among the hybrid models, ConViT ranked first, followed by CoaT (80.41%) and HuviT (76.35%), while MobileViT (73.75%) ranked last overall. Table 4 summarises the comparative metric profile across models, and Figure 2 shows the full ROC curves.

Because the differences between the leading architectures are of the order of one percentage point, the corresponding interval estimates are reported in Table 5. The 95% confidence interval for ConViT’s accuracy is 80.91–85.45% and that for ConvNeXt is 80.20–84.81%; the two overlap over almost their entire width, as do those of the next three models. In unpaired two-proportion tests with Holm correction (Table 5), the accuracy of ConViT was significantly higher than that of Swin Transformer, HuviT, ResNet-50, EfficientNet-B4 and MobileViT (adjusted *p* ≤ 0.009), but not significantly different from that of ConvNeXt, DeiT, CoaT, PVTv2 and XCiT (adjusted *p* ≥ 0.14). The leading models cannot be separated at this sample size. What Table 5 does establish is that a single point estimate at this sample size is compatible with a range of roughly ±2.3 percentage points, so the ordering of models separated by less than that should not be regarded as firmly established.

### 4.2. Class-Level Analysis of the Best-Performing Model

Table 6 presents per-class metrics for ConViT. The model achieved 83.0% precision and 82.2% recall for the benign class, and 83.6% precision and 84.3% recall for the malignant class. F1-scores were 82.7% and 83.9%, respectively, a difference of just 1.2 percentage points. This close alignment indicates that the model did not inflate aggregate accuracy by systematically favouring one class; its performance was balanced across both categories.

Expressed in the terms conventionally used for a diagnostic test, and taking malignancy as the positive condition, ConViT achieved a sensitivity of 84.33% (452/536; 95% CI 81.01–87.16), a specificity of 82.20% (411/500; 95% CI 78.61–85.30), a positive predictive value of 83.55% (452/541; 95% CI 80.19–86.43) and a negative predictive value of 83.03% (411/495; 95% CI 79.47–86.08); balanced accuracy was 83.26%. These quantities, which are the clinically interpretable ones for a benign-versus-malignant task, are set out in Table 7. Because the malignant fraction of the test set (51.7%) reflects the composition of the pooled research dataset rather than that of any clinical population, the two predictive values are properties of this cohort alone (Section 5). The precision, recall and F1-score reported in Table 4 for all eleven models are weighted averages over the two classes (weighted by class support) and are therefore not identical to the class-specific values given here.

### 4.3. Confusion Matrix

**Figure 3:**
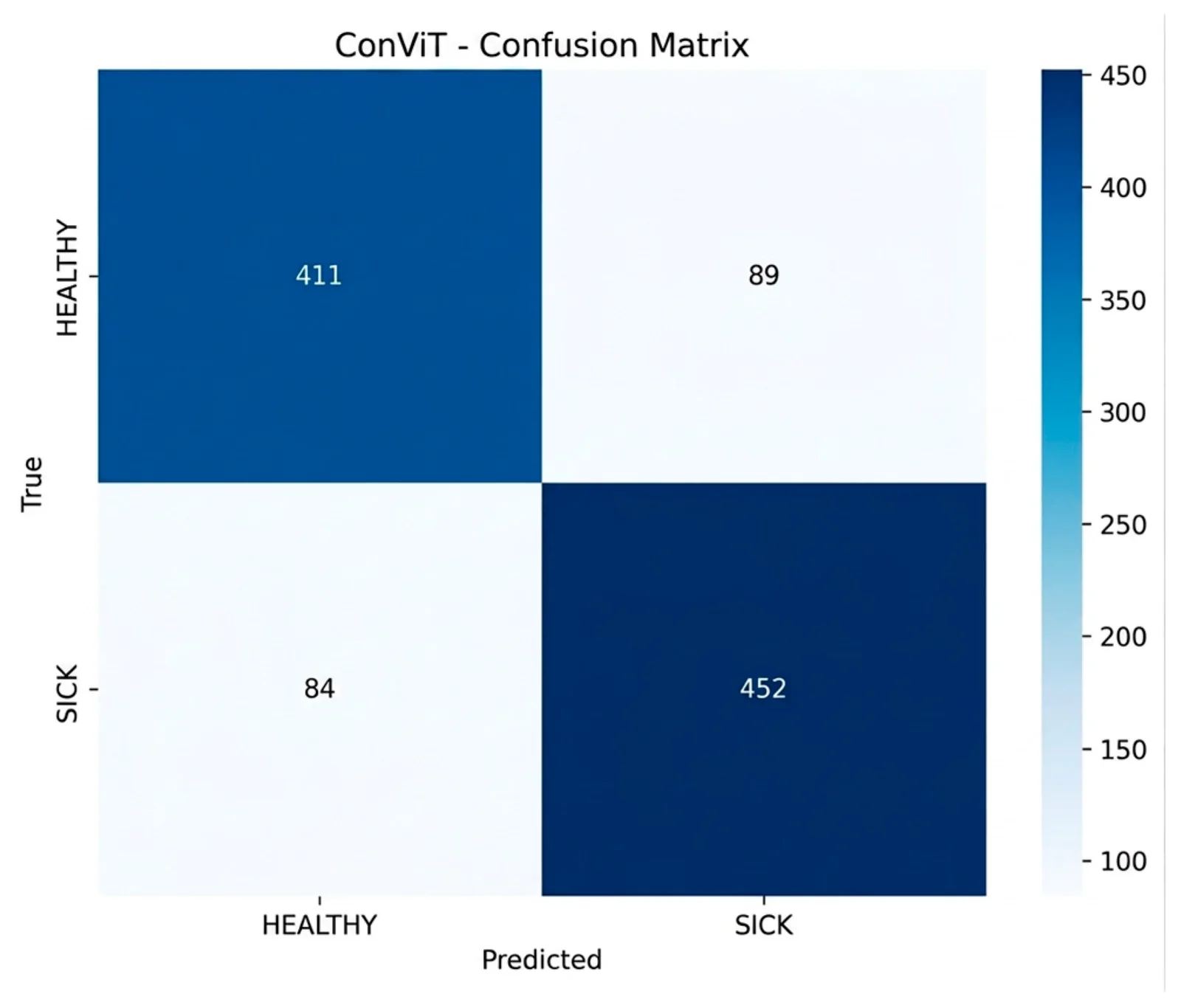
Confusion matrix for the ConViT model (*n* = 1036 test images). TP = 452, FN = 84 (malignant class); TN = 411, FP = 89 (benign class).

**Figure 4:**
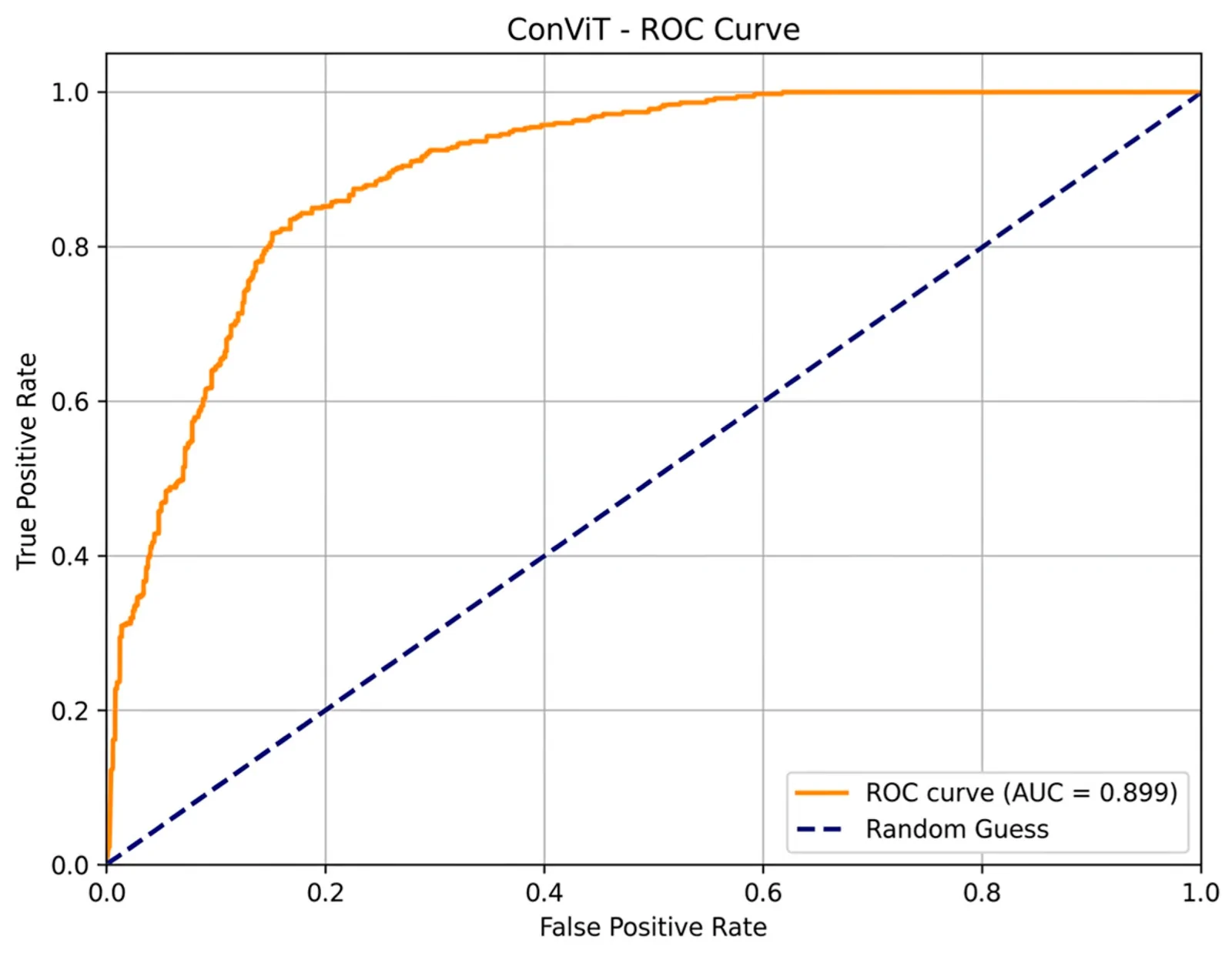
ROC curve for the ConViT model (AUC = 0.899). Proximity to the upper-left corner confirms high discriminative ability across all decision thresholds.

The confusion matrix (Figure 3) shows that ConViT correctly classified 452 of the 536 malignant cases (true positive rate 84.3%) and 411 of the 500 benign cases (true negative rate 82.2%). Eighty-four malignant cases were misclassified as benign (false negative), and 89 benign cases were misclassified as malignant (false positive). The difference between the two error counts is five images out of 1036, which is well within the range expected from sampling variation at this sample size; it is reported here as a descriptive observation and is not interpreted as evidence of a systematically cautious decision boundary. Any deliberate asymmetry between the two error types would have to be imposed explicitly, by moving the operating point along the ROC curve of Figure 4 to the sensitivity the clinical context requires, and no such threshold adjustment was performed in this study.

### 4.4. ROC Curve and AUC

The ROC curve for ConViT (Figure 4) remained close to the upper-left corner across all decision thresholds, confirming that the model’s discriminative ability was not concentrated at a single operating point. An AUC of 0.899 (95% CI 0.878–0.917) means that for a randomly chosen benign–malignant pair, the model assigned the higher probability to the malignant case approximately 90% of the time.

### 4.5. Training Dynamics

**Figure 5:**
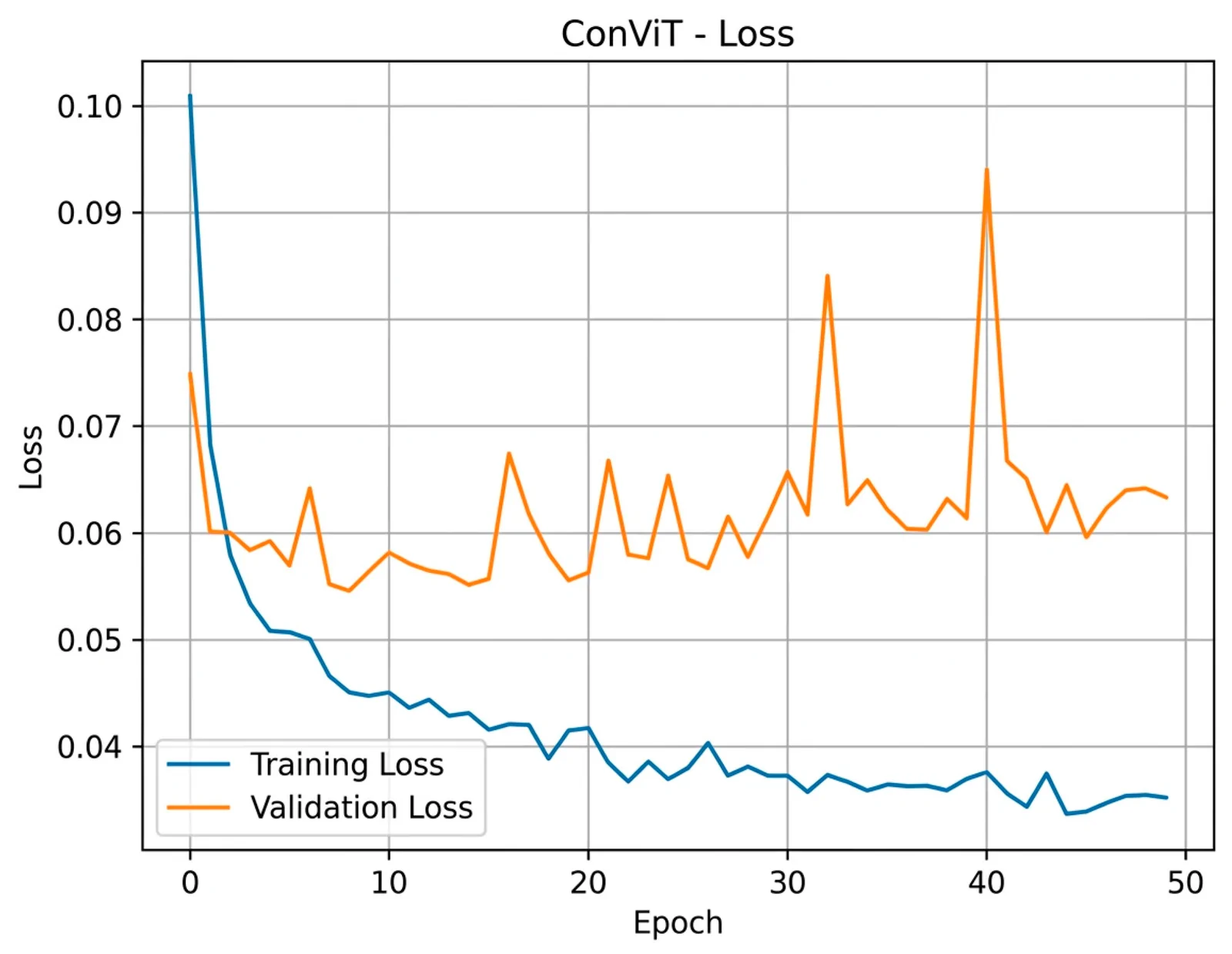
Epoch-by-epoch training (blue) and validation (orange) loss for ConViT over 50 epochs.

**Figure 6:**
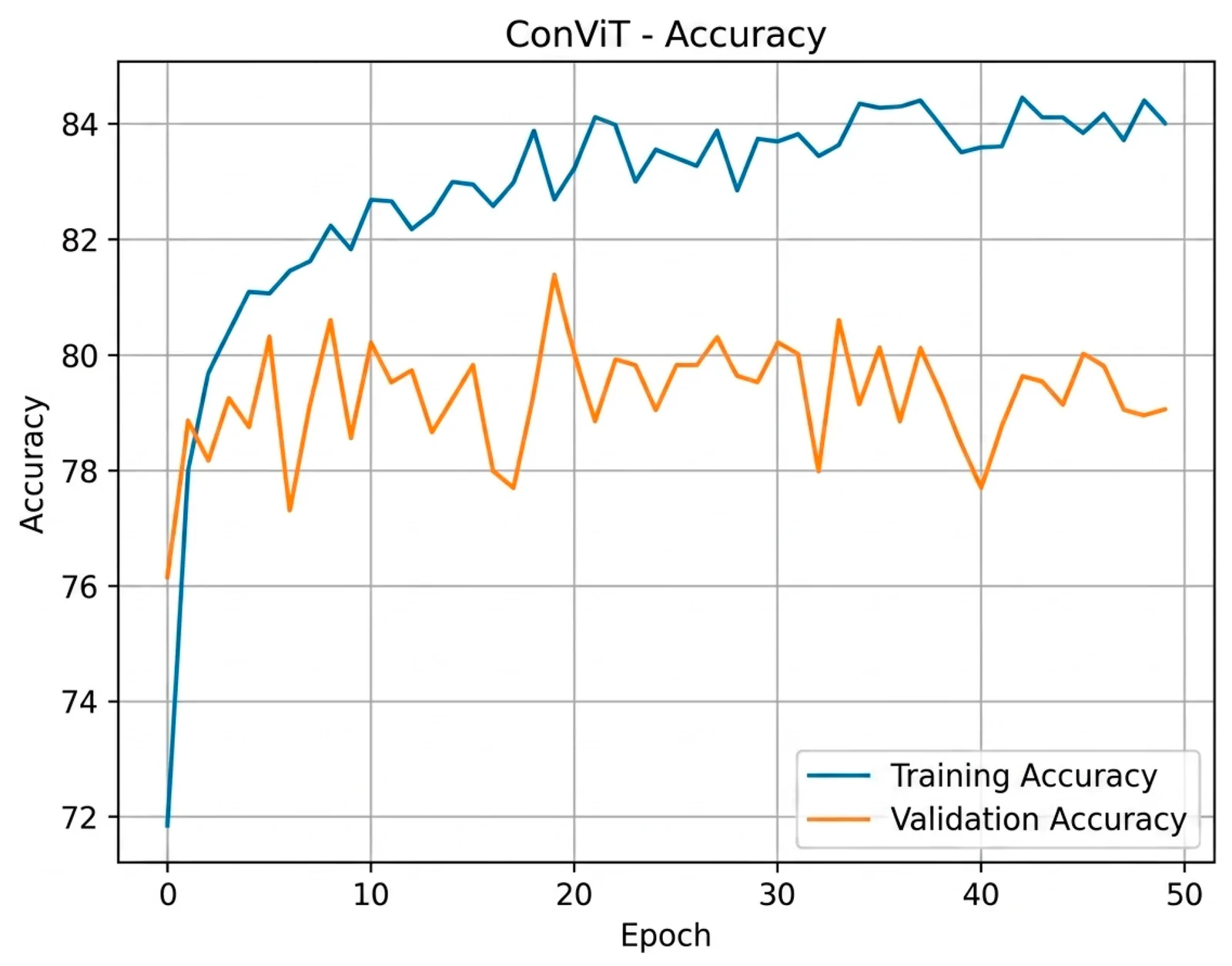
Epoch-by-epoch training (blue) and validation (orange) accuracy for ConViT over 50 epochs.

Figure 5 and Figure 6 display epoch-by-epoch training and validation loss and accuracy curves for ConViT over 50 epochs. Training loss fell from approximately 0.10 at the first epoch to below 0.04 by epoch 50. Validation loss settled in the range 0.055–0.067 after the first few epochs, with transient peaks of approximately 0.084 at epoch 32 and 0.094 at epoch 40 before returning to that range. Training accuracy rose sharply in the first ten epochs (from approximately 72% to approximately 83%), then improved gradually to above 84% at epoch 50. Validation accuracy tracked this trend, ranging between 78% and 82%. The moderate and non-diverging gap between training and validation metrics indicates adequate generalisation without overfitting.

### 4.6. Explainability Analysis

**Figure 7:**
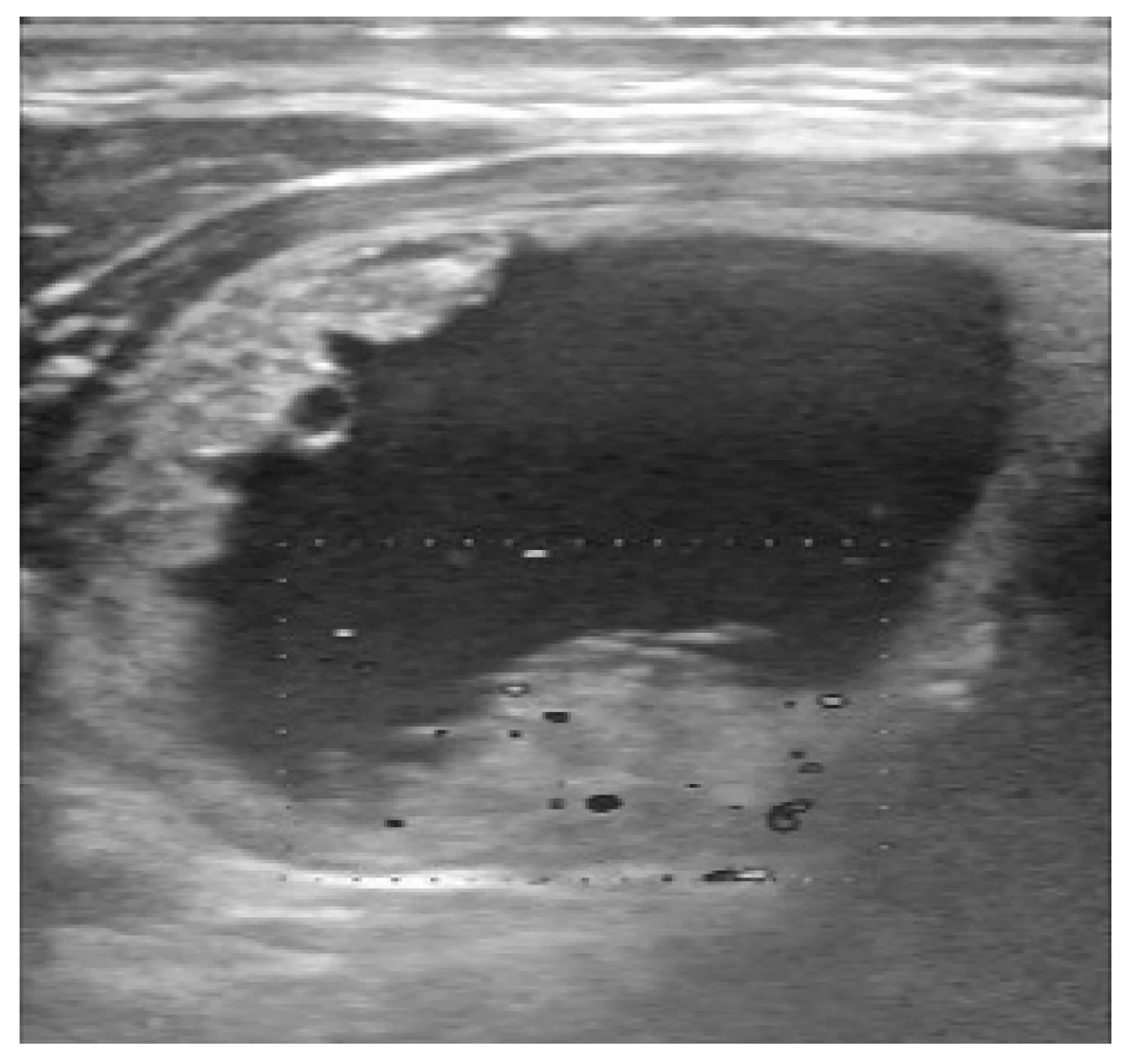
A representative benign thyroid ultrasound image classified for XAI analysis.

**Figure 8:**
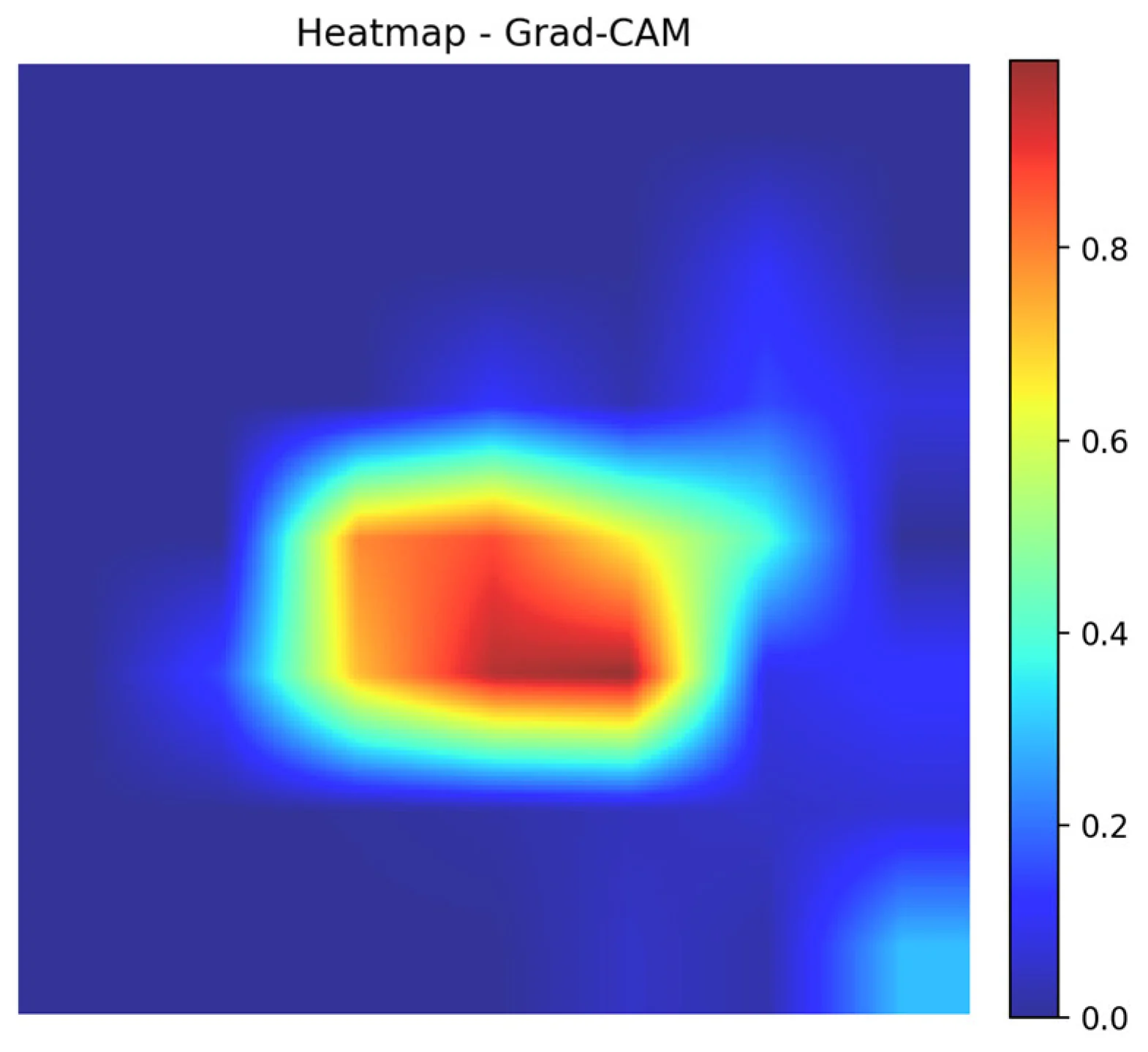
Grad-CAM heat map for the image in Figure 7. Warm colours indicate high-activation regions corresponding to nodule boundary and echogenicity patterns [42].

**Figure 9:**
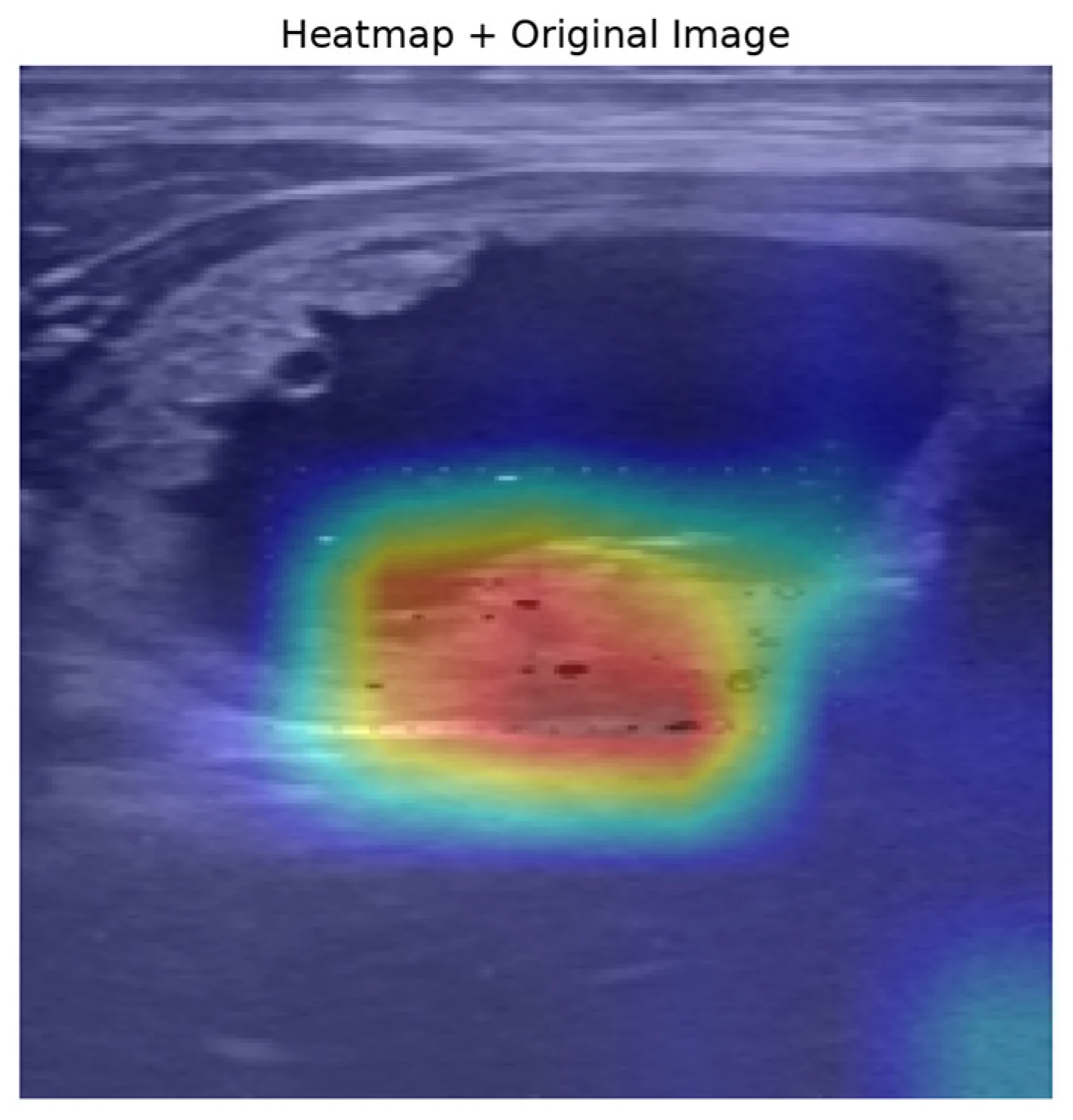
Superimposition of the Grad-CAM heat map on the original ultrasound image. Warm colors (red and yellow) indicate regions with the greatest contribution to the model’s prediction, whereas cool colors (blue) indicate regions with minimal contribution. Maximum activation: 0.994; activation area ratio: 0.309.

Grad-CAM heat maps for a representative benign case correctly classified are shown in Figure 7, Figure 8 and Figure 9 [42]. High-activation regions coincided with the nodule boundary and its internal echogenicity pattern, the same sonographic features radiologists assess when grading a nodule. Quantitative metrics for this case: maximum activation 0.994, mean activation 0.139, activation area ratio 0.309. The high maximum activation indicates that relevance was concentrated at a small number of peaks; an activation area ratio of 0.309 means that approximately 31% of the image was treated as informative. This represents a moderate rather than a tight spatial focus, and a single case cannot establish that the model attends to the lesion to the exclusion of surrounding tissue.

**Figure 10:**
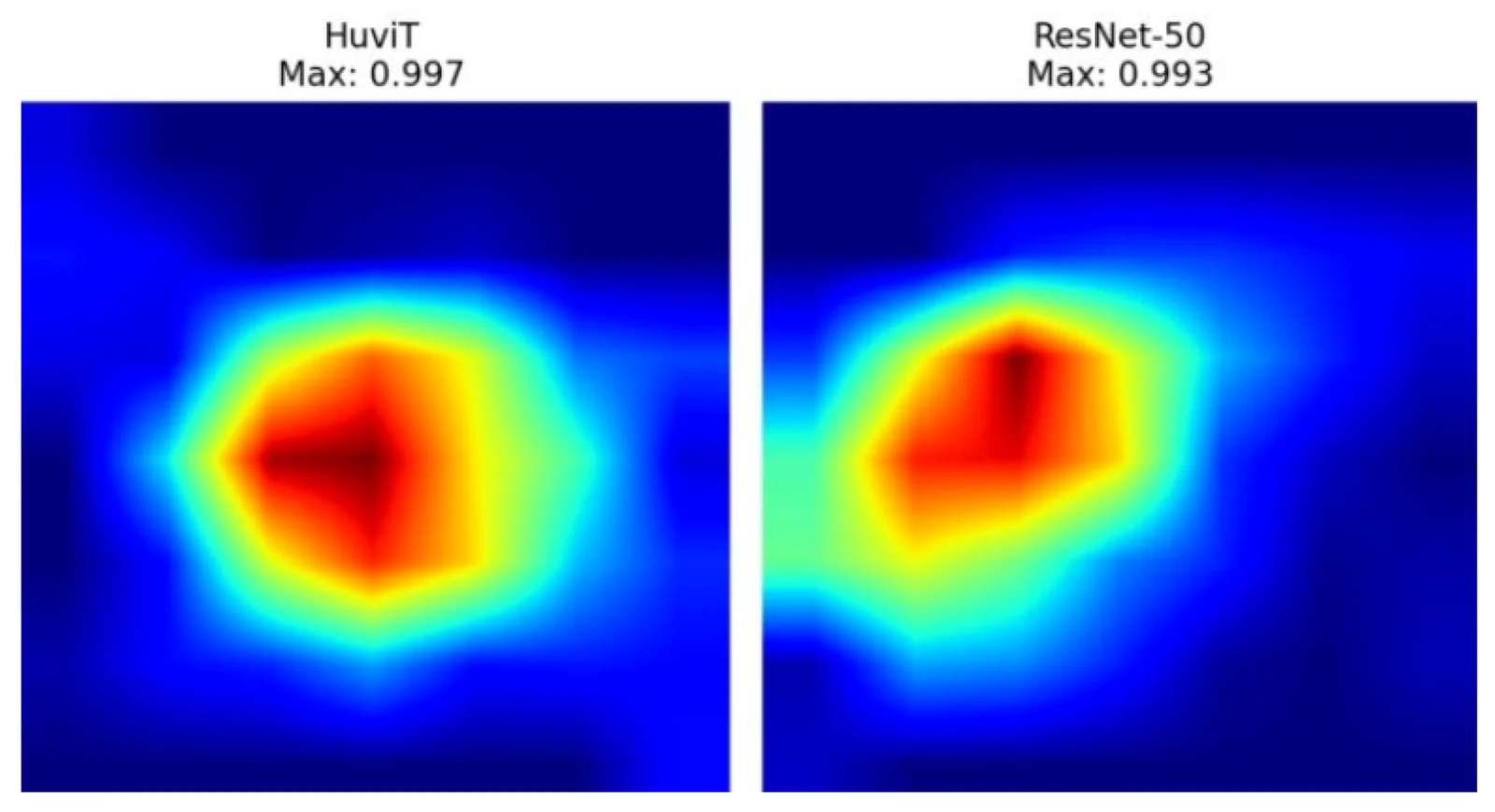
Multi-model Grad-CAM comparison on the same ultrasound image. Warm colors (red and yellow) indicate regions with the greatest contribution to the model’s prediction, whereas cool colors (blue) indicate regions with minimal contribution.

Figure 10 compares Grad-CAM maps from two models for the same image: HuviT (0.997) and ResNet-50 (0.993). Both models highlighted regions overlapping with the lesion, but the spatial spread differed. The high-activation region was the broadest and most central for HuviT, and obliquely oriented and intermediate in extent for ResNet-50. The two maximum values differ by only 0.004 and are not meaningfully different. These are descriptive observations from a single image and do not indicate that the models use different decision strategies. Whether such differences are systematic, and whether they reflect the architectural differences between the models, would require a quantitative comparison across multiple images, which is beyond the scope of the present study.

**Figure 11:**
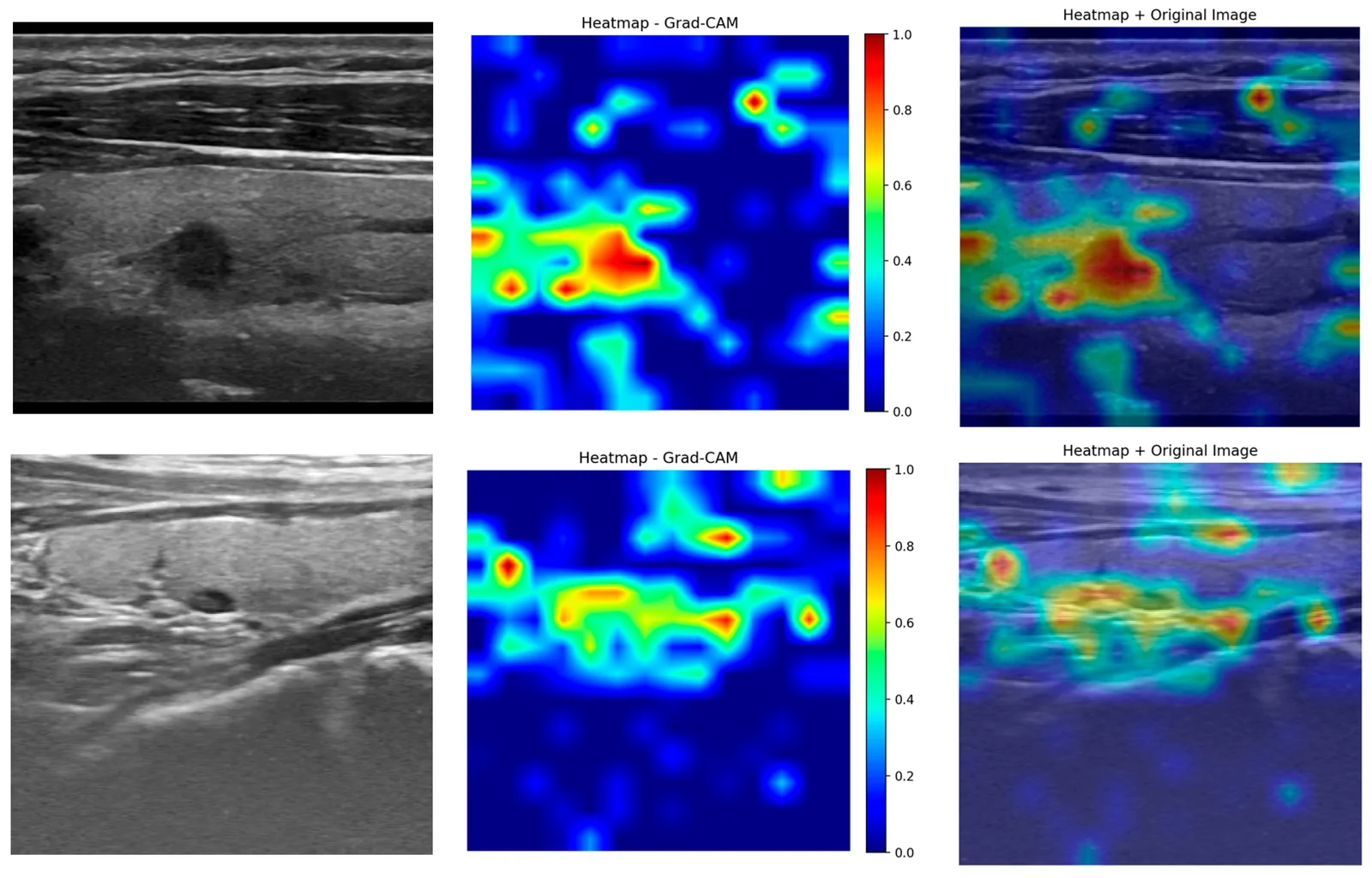
ConViT Grad-CAM analysis of two further test images (first image, top row; second image, bottom row). Warm colors (red and yellow) indicate regions with the greatest contribution to the model’s prediction, whereas cool colors (blue) indicate regions with minimal contribution. Columns, left to right: original ultrasound image; Grad-CAM heat map, with warm colours indicating high-activation regions; heat map superimposed on the original image.

Figure 11 shows ConViT Grad-CAM maps for two further test images. In the first (top row), the strongest activation overlapped the hypoechoic nodule, with additional local maxima of lower intensity in the parenchyma to the left of the nodule, in the superficial soft-tissue layers and along the image margins. In the second (bottom row), no single structure dominated: activation was distributed along a broad central band with several separate local maxima and was not clearly confined to the small dark focus visible in the original image. Together with Figure 7, Figure 8, Figure 9 and Figure 10, these examples indicate that the spatial concentration of Grad-CAM activation varies between images and is not always confined to the lesion.

## 5. Discussion

The central finding is that ConViT, which begins training with convolutional inductive biases and progressively shifts toward global self-attention through its gating mechanism, achieved the highest threshold-based scores among the eleven architectures in thyroid nodule classification [17]. Its balanced class-level performance (F1-scores of 82.7% for benign and 83.9% for malignant) is of particular clinical relevance. Both error types carry meaningful consequences: false negatives delay treatment for malignant lesions, while false positives expose patients with benign nodules to unnecessary biopsies. A model that distributes errors roughly equally across classes offers a more even starting point for threshold selection than one that concentrates most errors in a single direction. Whether any of these models is suitable as a screening adjunct is a separate question that the present, retrospective and prevalence-enriched evaluation cannot answer.

ConViT’s higher accuracy than the pure ViT models (statistically significant only against Swin Transformer in Table 5) is consistent with the behaviour expected from its GPSA design: early in training, attention acts like a convolution, exploiting the strong local regularities that CNNs encode efficiently; as training progresses, the gates open and the model extends its receptive field to global structure [17]. This progression allows the model to generalise well from approximately 4800 training images, a scale at which pure ViTs trained from scratch would likely underperform without extensive pretraining [8,10]. The gap between ConViT and the next best hybrid (CoaT, 80.41%) is compatible with the possibility that gradual inductive bias relaxation is more effective than static integration strategies that fix the local–global balance from the outset, although the confidence intervals of the two models overlap.

ConvNeXt’s strong performance merits attention. Achieving 82.63% accuracy and the highest overall ROC-AUC (90.02%) with a purely convolutional architecture shows that the gap between modernised CNNs and hybrid ViT models is smaller than a naive ‘CNN versus transformer’ framing implies. ConvNeXt’s design (large 7 × 7 depth-wise kernels, layer normalisation, and inverted bottleneck blocks) was borrowed directly from transformer design thinking, producing a convolutional model that captures longer-range dependencies than a standard ResNet [35]. The finding that its ROC-AUC marginally exceeded ConViT’s (0.9002 versus 0.8994) indicates that the two models rank cases comparably well, even though the threshold-based accuracy of ConvNeXt is slightly lower. This is consistent with analyses demonstrating ConvNeXt’s competitiveness in few-shot and medium-scale settings [43,44].

Taken as a group, however, the hybrid architectures did not outperform the other two paradigms. The four hybrids span almost the entire range of observed performance: ConViT first at 83.30%, CoaT fourth at 80.41%, HuviT eighth at 76.35% and MobileViT last of all eleven at 73.75%. The purely convolutional ConvNeXt was second on accuracy and first on ROC-AUC, and DeiT, the strongest pure ViT, achieved higher accuracy than three of the four hybrids. A claim of categorical superiority is therefore not sustainable, and the conclusion drawn here is about a mechanism rather than a label. The two best-performing architectures, ConViT and ConvNeXt, are precisely the two that most explicitly combine convolutional locality with long-range modelling: ConViT by initialising self-attention with a convolutional prior that relaxes as training proceeds [17], ConvNeXt by importing transformer design elements into a convolutional stack [35]. Architectures that instantiate the combination differently (MobileViT’s lightweight fusion optimised for on-device inference, HuviT’s untrained, randomly initialised encoder applied to features that the backbone has already compressed) did not benefit. These results are compatible with the view that the manner in which local and global modelling are combined matters more than whether a model is nominally described as hybrid. The comparison across the three paradigms is summarised in Table 8.

Among pure ViT architectures, Swin Transformer showed notably elevated precision (82.71%) relative to its accuracy (77.80%). Descriptively, this corresponds to comparatively few false positives at the cost of more missed cases; because no threshold adjustment was applied, the pattern is a property of the fitted decision function rather than a deliberate operating-point choice [19]. This may arise from its local-window attention design, which shares some locality bias with CNNs and may generate a decision function less sensitive to diffuse, globally distributed malignancy cues. For screening scenarios where specificity is the primary requirement, such a bias could be acceptable or even desirable, provided sensitivity losses are acceptable given the overall clinical workflow. DeiT’s relatively strong performance among pure ViTs can be attributed to its CNN-teacher distillation mechanism, which effectively infuses convolutional inductive biases into the student ViT and reduces its reliance on large training corpora [15,16].

HuviT, the novel architecture introduced here, achieved 76.35% accuracy, above ResNet-50, EfficientNet-B4 and MobileViT but below the other seven models. The current result represents a baseline rather than an optimised configuration: the projection and the transformer encoder were frozen at random initialisation, so that only the linear classifier was adapted to the data, and the transformer component adds global context modelling after spatial features are already compressed, which may limit its ability to reweight spatially distributed information. Future work should test whether training the projection and the transformer encoder, applying attention before major downsampling, using a more expressive backbone, or enabling full backbone fine-tuning with appropriate regularisation closes this gap.

The absolute performance reported here must also be read in light of the training protocol. All backbones were frozen to permit a fair comparison across architectures. Freezing converts each pretrained network into a fixed feature extractor, so that an ImageNet representation is applied unchanged to B-mode ultrasound images. The speckle texture, compressed dynamic range, single-channel acquisition, and near-absence of natural-object statistics in this modality differ markedly from the pretraining distribution. Early convolutional layers, which encode edges and oriented texture, transfer reasonably well, whereas later layers, which encode semantic categories of natural objects, transfer poorly. It is precisely these later layers that a frozen protocol prevents from adapting. Full fine-tuning would allow them to re-specialise on sonographic texture, lesion margins, and calcification, and in medical-imaging transfer learning it generally yields higher performance than linear probing.

The expected benefit of full fine-tuning, though, must be weighed against three considerations. First, with 4833 training images and backbone networks of roughly five to ninety million parameters, full fine-tuning may be prone to overfitting unless supported by layer-wise learning-rate decay, stronger regularisation, and early stopping; even then, both the expected gain and its variance may increase. Second, the optimal fine-tuning strategy may differ systematically between convolutional and transformer backbones. A single shared protocol, which is the very element that renders our eleven-way comparison interpretable, would therefore be difficult to maintain, and a fine-tuned comparison could to some extent confound architecture with tuning effort. Third, unfreezing only the final stage or block offers an intermediate solution that preserves most of the comparability while permitting limited domain adaptation; we consider this a more informative design for future work. Taken together, the frozen-backbone results reported here should be regarded as a conservative baseline obtained under a strictly uniform protocol, not as a ceiling on what these architectures can achieve on thyroid ultrasound.

The XAI analysis, based on a single representative case, suggested that the models examined activated radiologically plausible regions. Grad-CAM attention concentrated on nodule boundaries and internal echogenicity patterns rather than background structures [42], and the activation area ratio of approximately 31% indicates that relevance was distributed over a moderate portion of the image rather than the lesion alone. This interpretability property is a prerequisite for any future clinical evaluation: a model that achieves high aggregate accuracy by attending to irrelevant image features would not be trusted by clinicians regardless of its statistics. Multi-model heat map comparison suggested that HuviT’s heat map was distributed more broadly than that of ResNet-50, though whether this difference is systematic, and what it reflects, requires evaluation on multiple images with radiologist annotation.

A further consideration is the clinical interpretation of the predictive values. The consequence of the difference in malignancy prevalence between this cohort and routine practice (Section 3.1.3) can be quantified exactly. Holding ConViT’s sensitivity at 84.33% and specificity at 82.20% (both of which are properties of the classifier and do not depend on prevalence) and applying Bayes’ theorem, the positive predictive value falls from the 83.55% obtained on this test set to 45.53% at a malignancy prevalence of 15%, to 34.49% at 10% and to 19.96% at 5%, while the negative predictive value rises from 83.03% to between 96.75% and 99.01% over the same range (Table 9). At a prevalence typical of unselected nodules, therefore, roughly two of every three images the model flags as malignant would be benign. This is arithmetic rather than a new experiment, and it is reported here because the headline accuracy of 83.30% would otherwise invite a reading that the data do not support: the model’s rule-out performance transfers well to a lower-prevalence setting, whereas its rule-in performance does not.

Several limitations merit acknowledgment, and four of them are material to the interpretation of the results. First, the pooled dataset draws on two sources rather than on a multi-centre consortium: TN5000 was collected at a single tertiary oncology centre on two families of GE scanners, and the provenance, acquisition protocol and patient demographics of the second source are undocumented, so generalisability cannot be assumed without external and prospective validation. Second, because every malignant image comes from TN5000 while 57.2% of the benign images come from the second source, image source is partly associated with the class label; a source-only classifier would reach 79.35% accuracy on these data, and until a source-stratified analysis has been performed the possibility that part of the reported performance derives from source-specific appearance rather than from lesion morphology cannot be excluded. Third, the labels rest on two different reference standards (FNA biopsy with pathological confirmation for TN5000, and an undocumented standard for the added benign images), so the benign class is not uniformly verified. Fourth, partitioning was performed at the image level because patient and nodule identifiers are not released with either dataset; although the TN5000 curation retained only one representative image per imaging perspective per patient, residual correlation between training and test images cannot be ruled out, and the reported performance may therefore be modestly optimistic relative to a strict patient-level evaluation. Freezing backbone weights during training may have prevented the full adaptation of ImageNet features to thyroid ultrasound characteristics; full fine-tuning with appropriate regularisation could improve results across all architectures. The fixed hyperparameter protocol ensures fair comparison but does not identify the individually optimal settings for each architecture, and targeted hyperparameter search could narrow or widen the observed performance gaps. In particular, the projection and transformer encoder of HuviT were not trained in the present study, and ablation tests on its transformer hyperparameters have not yet been completed, because these experiments are lengthy and are still in progress. The XAI findings are based on a single representative case and a limited multi-model comparison; a systematic study correlating model attention maps with structured radiologist annotation would be needed to draw firm conclusions about clinical interpretability.

Three further limitations follow from what the public data do not contain. First, performance could not be stratified by nodule composition (solid, cystic, mixed, calcified or anechoic) because neither source distributes these descriptors, so the behaviour of the models on precisely the sonographic subgroups that are hardest for a human reader remains unknown, as does their behaviour in multinodular glands. Second, the models were not benchmarked against a structured ultrasound risk-stratification score such as the European Thyroid Association Thyroid Imaging Reporting and Data System (EU-TIRADS) [45] or the American College of Radiology TI-RADS [46], nor against the cytological categories of the 2017 Bethesda System for Reporting Thyroid Cytopathology [47], because neither dataset releases the ultrasound feature scores or the cytology reports from which those categories are derived; TN5000 states explicitly that pathology numbers and biopsy detail were erased at anonymisation [30]. It is worth being precise about what is and is not missing here: the class labels used in this study are themselves anchored in FNA and pathology rather than in a TI-RADS surrogate, so the models are already evaluated against a pathology-derived reference standard. What is absent is the comparison with the radiologist’s score, not the pathological ground truth itself. Third, and consequently, no reader study was performed, so the clinically decisive question (whether these models add to, or merely reproduce, expert assessment) is untouched by the present data. A prospective study collecting paired EU-TIRADS scores [45], Bethesda categories [47] and model outputs on the same nodules is the appropriate next step and is proposed in Section 6. Such a design would also allow the comparison that matters most clinically, namely whether model output changes the fine-needle aspiration decision that EU-TIRADS or ACR TI-RADS would otherwise dictate for a given nodule size and risk category [32,45,46].

## 6. Conclusions

This study compared eleven deep learning architectures for thyroid nodule ultrasound classification across three paradigms. ConViT, which integrates convolutional inductive biases with global self-attention through a gating mechanism, ranked highest on accuracy, precision, recall and F1-score among the eleven architectures, although its margin over the best-performing convolutional model was within the range of sampling variation and that model attained the higher ROC-AUC. The results do not establish a general superiority of hybrid CNN–Transformer architectures over pure CNN or ViT models: the four hybrids evaluated here occupy both the top and the bottom of the ranking, and a purely convolutional model attained the highest ROC-AUC. They do, however, suggest the potential of specific designs. ConViT, and more tentatively the design principle it shares with ConvNeXt, combined convolutional locality with long-range modelling effectively under a strictly uniform protocol. The balanced per-class error distribution and the anatomically plausible Grad-CAM maps are encouraging, but their clinical relevance remains to be established: the attention maps were not validated against radiologist annotation, the models were not compared with structured ultrasound risk stratification or with human readers, the evaluation was retrospective and internal, and the partial association between image source and class label in the pooled dataset still needs to be excluded as a contributing factor. These architectures should therefore be regarded as promising candidates for further evaluation, in particular prospective external validation. ConvNeXt demonstrated that carefully designed CNN architectures remain highly competitive, particularly in terms of discriminative ability as measured by ROC-AUC. HuviT provides a modular, transfer-learning-friendly baseline whose performance can likely be improved through architectural refinements including higher-resolution transformer integration and targeted fine-tuning; its present result should be read in the light of the fact that, alone among the eleven models, its distinguishing components (the projection and the transformer encoder) had neither pretrained weights nor training and were frozen at random initialisation. Future work should prioritise external validation with prospectively collected data and patient-level partitioning, architecture-specific hyperparameter optimisation (including training of the HuviT projection and encoder and ablation of its transformer hyperparameters), expansion to multi-class TI-RADS grading, subgroup analysis by sonographic nodule composition and in multinodular glands, direct comparison against EU-TIRADS [45] and ACR TI-RADS [46] risk stratification and against cytological Bethesda categories [47], and controlled reader studies evaluating whether model-generated attention maps improve radiologist diagnostic consistency.

## Figures and Tables

**Table 1:** Taxonomy of hybrid deep learning architectures: structural features, advantages, limitations, and representative application domains.

Hybrid Model	Structural Feature	Advantages	Limitations	Application Domains
Sequential Hybrid	Outputs transferred serially across stages	Modularity, task-specific optimisation, high accuracy	Error propagation, high resource demand, structural complexity	Medical imaging, NLP, learning analytics
Parallel Hybrid	Simultaneous, independent model execution with decision fusion	Model diversity, reduced overfitting, decision fusion flexibility	High compute cost, output combination complexity	Biomedical analysis, NLP, image processing
Transformational Hybrid	Each component semantically re-encodes the data	Deep abstraction, contextual representation, versatility	Reduced interpretability, potential information loss	Genomics, NLP, learning analytics
CNN-based	CNN feature extraction + ML/DL classifier	Rich feature extraction, flexible integration	Computation-intensive, large data requirements	Tumour analysis, agricultural disease detection
ViT-based	ViT feature extraction + CNN or ML decision component	Global context learning, strong performance with limited data	High data/resource demands, limited interpretability	Medical imaging, quality control, defence
GNN-based	Graph-structured data encoding combined with other architectures	Relational learning, heterogeneous data analysis	Graph construction overhead, integration complexity	Molecular analysis, social networks, recommender systems
RNN/LSTM-based	Temporal data via LSTM; spatial features handled separately	Temporal pattern learning, contextual sequence modelling	Computational cost for long sequences, hyperparameter sensitivity	Learning analytics, financial forecasting, signal processing
Hierarchical Hybrid	Layered architecture; each tier addresses a specific sub-task	Multi-level task decomposition, task specialisation, flexibility	Design complexity, lower-tier error propagation	Medical diagnosis, educational data analysis, industrial automation

Abbreviations: DL, deep learning; GNN, graph neural network; LSTM, long short-term memory; ML, machine learning; NLP, natural language processing; RNN, recurrent neural network.

**Table 2:** Shared training hyperparameters applied identically to all eleven architectures.

Parameter	Value/Setting	Description
Learning Rate	0.001	Controls step size during gradient-based optimisation
Batch Size	32	Number of samples processed per iteration
Epochs	50	Number of full passes through the training set
Optimiser	AdamW	Adaptive moment estimation with decoupled weight decay
Loss Function	Focal Loss	Focal loss (α = 1, γ = 2)
Weight Decay	0.01	Decoupled weight decay coefficient (PyTorch (v2.7.1+cu118) AdamW default)
Optimiser Betas	0.9, 0.999	Exponential decay rates for the first- and second-moment estimates in AdamW
Random Seed	42	Fixed seed for reproducibility

Abbreviations: AdamW, Adam optimiser with decoupled weight decay.

**Table 3:** Total and trainable parameter counts for the eleven evaluated architectures under the frozen-backbone protocol.

Model	Model Identifier	Total Params (M)	Trainable Params (M)	Trainable Components
ResNet-50	resnet50	23.51	0.0041	Final linear classifier only: 2 × (2048 + 1) = 4098
EfficientNet-B4	efficientnet_b4	17.55	0.0036	Final linear classifier only: 2 × (1792 + 1) = 3586
ConvNeXt	convnext_base	87.57	0.0041	Classifier block only: LayerNorm2d (2 × 1024) + Linear 2 × (1024 + 1) = 4098
Swin Transformer	swin_tiny_patch4_window7_224	27.52	0.0015	Final linear head only: 2 × (768 + 1) = 1538
DeiT	deit_base_patch16_224	85.80	0.0015	Final linear head only: 2 × (768 + 1) = 1538
PVTv2	pvt_v2_b2	24.85	0.0010	Final linear head only: 2 × (512 + 1) = 1026
XCiT	xcit_small_12_p8_224	25.83	0.0008	Final linear head only: 2 × (384 + 1) = 770
ConViT	convit_base	85.77	0.0015	Final linear head only: 2 × (768 + 1) = 1538
CoaT	coat_lite_mini	10.50	0.0010	Final linear head only: 2 × (512 + 1) = 1026
MobileViT	mobilevit_s	4.94	0.0013	Final linear classifier only: 2 × (640 + 1) = 1282
HuviT	resnet50 backbone + custom Transformer encoder	39.22	0.0010	Final linear classifier only: 2 × (512 + 1) = 1026

**Table 4:** Classification performance of eleven deep learning architectures on the held-out thyroid ultrasound test set (*n* = 1036 images).

Model	Accuracy	Precision	Recall	F1-Score	ROC-AUC	ROC-AUC 95% CI
ConViT	0.8330	0.8330	0.8326	0.8330	0.8994	0.878–0.917
ConvNeXt	0.8263	0.8312	0.8238	0.8251	0.9002	0.879–0.918
DeiT	0.8118	0.8134	0.8101	0.8112	0.8910	0.869–0.910
CoaT	0.8041	0.8084	0.8016	0.8028	0.8785	0.855–0.898
PVTv2	0.8002	0.8015	0.7986	0.7996	0.8753	0.852–0.895
XCiT	0.7954	0.7964	0.7938	0.7948	0.8604	0.836–0.882
Swin Transformer	0.7780	0.8271	0.7709	0.7673	0.8849	0.862–0.904
HuviT	0.7635	0.7709	0.7601	0.7608	0.8386	0.812–0.861
ResNet-50	0.7568	0.7711	0.7602	0.7548	0.8771	0.854–0.897
EfficientNet-B4	0.7490	0.7530	0.7462	0.7471	0.8105	0.783–0.835
MobileViT	0.7375	0.7379	0.7360	0.7369	0.8118	0.784–0.836

Precision, recall and F1-score are weighted averages over the two classes (weighted by class support); ROC-AUC is computed from the continuous predicted scores. The 95% confidence intervals (CIs) for ROC-AUC were computed with Newcombe’s score-type method.

**Table 5:** Accuracy of the eleven architectures on the held-out test set with two-sided 95% Wilson score confidence intervals (*n* = 1036). *p*-values were calculated using unpaired two-proportion z-tests against ConViT with Holm correction.

Model	Correct/n	Accuracy (%)	95% CI (%)	Paradigm	Holm-Adjusted *p* vs. ConViT
ConViT	863/1036	83.30	80.91–85.45	Hybrid	Reference
ConvNeXt	856/1036	82.63	80.20–84.81	CNN	0.68
DeiT	841/1036	81.18	78.68–83.44	ViT	0.41
CoaT	833/1036	80.41	77.88–82.71	Hybrid	0.26
PVTv2	829/1036	80.02	77.48–82.34	ViT	0.21
XCiT	824/1036	79.54	76.97–81.88	ViT	0.14
Swin Transformer	806/1036	77.80	75.17–80.22	ViT	0.009
HuviT	791/1036	76.35	73.67–78.84	Hybrid	<0.001
ResNet-50	784/1036	75.68	72.97–78.19	CNN	<0.001
EfficientNet-B4	776/1036	74.90	72.17–77.45	CNN	<0.001
MobileViT	764/1036	73.75	70.98–76.33	Hybrid	<0.001

**Table 6:** Class-level performance metrics for the ConViT model on the test set (benign: *n* = 500; malignant: *n* = 536).

Class	Precision	Recall	F1-Score	Sample Count
Benign	0.830	0.822	0.827	500
Malignant	0.836	0.843	0.839	536

**Table 7:** Diagnostic performance of the ConViT model on the held-out test set, with malignancy as the positive condition (*n* = 1036; 536 malignant, 500 benign).

Measure	Count	Estimate (%)	95% CI (%)	Comment
Sensitivity (recall, malignant)	452/536	84.33	81.01–87.16	Proportion of malignant images correctly identified
Specificity (recall, benign)	411/500	82.20	78.61–85.30	Proportion of benign images correctly identified
Positive predictive value	452/541	83.55	80.19–86.43	Prevalence-dependent; cohort-specific
Negative predictive value	411/495	83.03	79.47–86.08	Prevalence-dependent; cohort-specific
Balanced accuracy		83.26		Mean of sensitivity and specificity
Overall accuracy	863/1036	83.30	80.91–85.45	All correctly classified images

Intervals are two-sided 95% Wilson score intervals.

**Table 8:** Summary of the comparison across the three architectural paradigms on the held-out test set (*n* = 1036).

Paradigm	Architectures	Accuracy Range (%)	ROC-AUC Range	Summary of Comparative Behaviour
CNN	ResNet-50, EfficientNet-B4, ConvNeXt	74.90–82.63	0.8105–0.9002	Widest ROC-AUC range; ConvNeXt best overall on ROC-AUC
ViT	Swin, DeiT, PVTv2, XCiT	77.80–81.18	0.8604–0.8910	Narrowest accuracy range; no model in the top two
Hybrid	ConViT, CoaT, MobileViT, HuviT	73.75–83.30	0.8118–0.8994	Contains both the best and the worst model of the study

Ranges are the minimum and maximum values observed within each paradigm in Table 4. The three paradigms contain three, four and four architectures respectively, so the ranges are not directly comparable as estimates of central tendency and are reported to show overlap rather than to rank the paradigms.

**Table 9:** Predictive values of the ConViT classifier as a function of malignancy prevalence, at fixed sensitivity (84.33%) and specificity (82.20%).

Malignancy Prevalence (%)	Positive Predictive Value (%)	Negative Predictive Value (%)	Setting
5	19.96	99.01	Screening/unselected nodules (lower bound of the usual range)
10	34.49	97.93	Unselected nodules referred for ultrasound
15	45.53	96.75	Unselected nodules (upper bound of the usual range)
30	67.00	92.45	Enriched referral population
51.7	83.55	83.03	The present test set (research cohort)

Values are obtained from Bayes’ theorem using the sensitivity and specificity reported in Table 7.

## Data Availability

The datasets used in this study were obtained from publicly available datasets reported in previously published studies: refs. [30,31].

## Supplementary Materials

The following supporting information can be downloaded at https://www.mdpi.com/article/10.3390/diagnostics16213640/s1: Figures S1–S8, screen captures of the Streamlit-based application illustrating, respectively, Figure S1. data loading, Figure S2. preprocessing, Figure S3. augmentation, Figure S4. partitioning, Figure S5. model training, Figure S6. evaluation, Figure S7. explainability, and Figure S8. results and reporting.
